# COVID-19 Vaccine Provider Access and Vaccination Coverage Among Children Aged 5–11 Years — United States, November 2021–January 2022

**DOI:** 10.15585/mmwr.mm7110a4

**Published:** 2022-03-11

**Authors:** Christine Kim, Randy Yee, Roma Bhatkoti, David Carranza, Danielle Henderson, Sachiko A. Kuwabara, James Phillip Trinidad, Sandra Radesky, Allen Cohen, Tara M. Vogt, Zachary Smith, Chris Duggar, Kevin Chatham-Stephens, Christina Ottis, Krista Rand, Travis Lim, Alice F. Jackson, Donald Richardson, Aaron Jaffe, Rachael Lubitz, Ryan Hayes, Aran Zouela, Deborah L. Kotulich, Patrick N. Kelleher, Angela Guo, Satish K. Pillai, Anita Patel

**Affiliations:** ^1^Epidemic Intelligence Service, CDC; ^2^CDC COVID-19 Emergency Response Team; ^3^Countermeasures Acceleration Group, U.S. Department of Health and Human Services Coordination Operations and Response Element, Washington, DC; ^4^Johns Hopkins University Applied Physics Laboratory, Laurel, Maryland; ^5^Palantir Technologies, Palo Alto, California; ^6^Optum Serve, Falls Church, Virginia; ^7^Strategic Innovative Solutions, LLC, Clearwater, Florida.

On October 29, 2021, the Pfizer-BioNTech pediatric COVID-19 vaccine received Emergency Use Authorization for children aged 5–11 years in the United States.^†^ For a successful immunization program, both access to and uptake of the vaccine are needed. Fifteen million doses were initially made available to pediatric providers to ensure the broadest possible access for the estimated 28 million eligible children aged 5–11 years, especially those in high social vulnerability index (SVI)^§^ communities. Initial supply was strategically distributed to maximize vaccination opportunities for U.S. children aged 5–11 years. COVID-19 vaccination coverage among persons aged 12–17 years has lagged (*1*), and vaccine confidence has been identified as a concern among parents and caregivers (*2*). Therefore, COVID-19 provider access and early vaccination coverage among children aged 5–11 years in high and low SVI communities were examined during November 1, 2021–January 18, 2022. As of November 29, 2021 (4 weeks after program launch), 38,732 providers were enrolled, and 92% of U.S. children aged 5–11 years lived within 5 miles of an active provider. As of January 18, 2022 (11 weeks after program launch), 39,786 providers had administered 13.3 million doses. First dose coverage at 4 weeks after launch was 15.0% (10.5% and 17.5% in high and low SVI areas, respectively; rate ratio [RR] = 0.68; 95% CI = 0.60–0.78), and at 11 weeks was 27.7% (21.2% and 29.0% in high and low SVI areas, respectively; RR = 0.76; 95% CI = 0.68–0.84). Overall series completion at 11 weeks after launch was 19.1% (13.7% and 21.7% in high and low SVI areas, respectively; RR = 0.67; 95% CI = 0.58–0.77). Pharmacies administered 46.4% of doses to this age group, including 48.7% of doses in high SVI areas and 44.4% in low SVI areas. Although COVID-19 vaccination coverage rates were low, particularly in high SVI areas, first dose coverage improved over time. Additional outreach is critical, especially in high SVI areas, to improve vaccine confidence and increase coverage rates among children aged 5–11 years.

To facilitate equitable access to pediatric COVID-19 vaccine for all children aged 5–11 years, doses were distributed through vaccination partners from state and local health departments, including Vaccines for Children^¶^ (VFC) program providers and other providers (jurisdictions),** the Federal Retail Pharmacy Program^††^ (FRPP), and federal entities.^§§^ Vaccination program operations considered vaccine supply, packaging, shelf life, site training, ability to vaccinate children aged 5–11 years, demand, and equity in the distribution strategy and selection of initial vaccine providers.^¶¶^

COVID-19 vaccine administration data reported to the U.S. Department of Health and Human Services (HHS) and CDC by partners via immunization information systems, the Vaccine Administration Management System, or direct data submission, and county-level SVI data were analyzed.*** Active providers were defined as those who received shipments or administered ≥1 BNT162b2 (Pfizer-BioNTech) pediatric COVID-19 vaccine dose in the preceding 28 days or reported inventory in the preceding 7 days. COVID-19 vaccination coverage was defined as the number of children who received ≥1 dose, or who received 2 doses (primary series completion), during November 1, 2021–January 18, 2022, divided by county population totals for those aged 5–11 years. Data reported to CDC by January 28, 2022, were included in the analysis. Total county pediatric population denominators used to create vaccination coverage estimates were obtained from the U.S. Census Bureau 2019 population estimates.^†††^ WorldPop 2020 data were used for the mapped population.^§§§^ SVI data were obtained from CDC’s 2018 SVI database. County-level SVI rankings were used; geospatial analysis used census tract–level SVI.^¶¶¶^ Provider county was used to determine provider SVI, and recipients’ county was used for vaccine recipient SVI. SVI rank cutoffs of 0–0.5 for low and >0.5–1 for high SVI were used.****

The number and geographic distribution of active providers by November 29, 2021, and January 18, 2022 (4 and 11 weeks, respectively, after the COVID-19 vaccination program launch on November 1, 2021) were assessed for children aged 5–11 years by SVI area. Data are presented at 4 weeks to illustrate the situation during the early program launch, and at 11 weeks, after peak demand, and during which the most recent data were available. The proportion of children who lived within 5 miles of an active provider was estimated, and the percentage of doses administered and total vaccination coverage rates by 4 and 11 weeks after the program launch were calculated by high and low SVI areas. RRs were calculated with corresponding 95% CIs to evaluate coverage rates between high and low SVI areas with generalized estimating equation models using binomial regression and log link.^††††^ Statistical analyses were conducted using Stata (version 16; StataCorp); CIs that excluded 1.0 were considered statistically significant. Maps were generated using QGIS (version 3.24; QGIS Association). This activity was reviewed by CDC and was conducted consistent with applicable federal law and CDC policy.^§§§§^

By 4 and 11 weeks after launch of the pediatric COVID-19 vaccination program, there were 38,732 and 39,786 active providers, respectively (Table) (Figure 1). Overall, and in high SVI areas, 92% of children aged 5–11 years lived within 5 miles (8 km) of an active provider, and in low SVI areas, 89% of children aged 5–11 years lived within 5 miles (8 km) of an active provider (Figure 2). Across states, 73%–100% of children aged 5–11 years lived within 5 miles (8 km) of an active provider, overall and in high SVI areas. By 11 weeks after launch, 54.0% of active providers were in high SVI areas, 44.6% and 32.7% of whom were FRPP and VFC providers, respectively (Supplementary Table 1, https://stacks.cdc.gov/view/cdc/114935). At 4 and 11 weeks after launch, 39.3% and 43.1% of first doses were administered in high SVI areas, respectively (Supplementary Table 2, https://stacks.cdc.gov/view/cdc/114935).

**TABLE:** Active pediatric COVID-19 vaccine providers* for children aged 5–11 years at 4 and 11 weeks after launch of pediatric COVID-19 vaccination program,^†^ by county-level social vulnerability index^§^ and provider type — United States, November 1, 2021–January 18, 2022

Characteristic	No. (%) of providers	
	4 weeks after program launch	11 weeks after program launch
**SVI area**		
High SVI areas	20,625 (53.2)	21,480 (54.0)
Low SVI areas	18,092 (46.7)	18,293 (46.0)
**Total**	**38,732 (100)**	**39,786 (100)**
**Provider type**		
VFC	12,171 (31.4)	13,164 (33.1)
FRPP	17,064 (44.1)	17,581 (44.2)
Federal entities	923 (2.4)	854 (2.1)
Other	8,574 (22.1)	8,187 (20.6)

**Abbreviations:** FRPP = Federal Retail Pharmacy Program; SVI = social vulnerability index; VFC = Vaccines for Children.

* Active providers included from 62 jurisdiction partners, 21 pharmacy partners, and five federal entity partners.

^†^ As of November 1, 2021.

^§^ SVI is composed of ranks from lowest (0) to highest (1) vulnerability. Rank cutoffs of 0–0.5 for low SVI and >0.5–1 for high SVI were used. https://www.atsdr.cdc.gov/placeandhealth/svi/index.html

**FIGURE 1:**
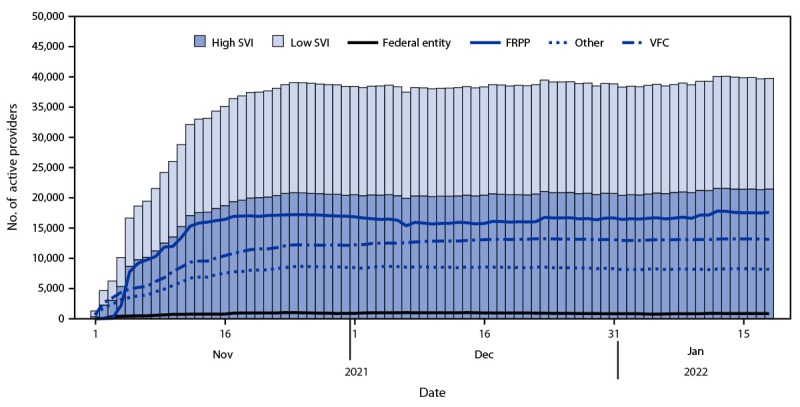
Active pediatric COVID-19 vaccine providers,* by social vulnerability index,^†^ provider type, and date — United States, November 1, 2021–January 18, 2022 **Abbreviations:** FRPP = Federal Retail Pharmacy Program; SVI = social vulnerability index; VFC = Vaccines for Children. * Active providers included 62 jurisdiction partners, 21 pharmacy partners, and five federal entity partners. ^†^ SVI is a composite measure of resilience, and includes socioeconomic status, household composition and disability, minority status and English language facility, and housing type and transportation. SVI is composed of ranks from lowest (0) to highest (1) vulnerability. Rank cutoffs of 0–0.5 for low SVI and >0.5–1 for high SVI were used. https://www.atsdr.cdc.gov/placeandhealth/svi/index.html

**FIGURE 2:**
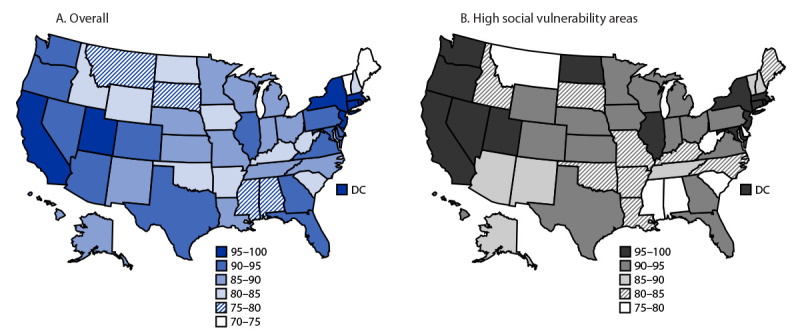
Percentage* of children aged 5–11 years living within 5 miles (8 km) of an active pediatric COVID-19 vaccine provider,^†^ by state, 4 weeks after pediatric vaccination program launch, overall (A) and in high social vulnerability^§^ areas (B) — United States, November 1, 2021–January 18, 2022 **Abbreviations:** DC = District of Columbia; SVI = social vulnerability index. * Maps depict geographical distance of the population to a provider offering vaccine for children aged 5–11 years. States where vaccine access is lower (<81% of the population lives within a within a 5 mile [8 km] radius of a vaccination site) might be because of various factors including rurality, lower number of active jurisdictional providers, and in rare instances jurisdictional plans had lower number of vaccination providers but still had high vaccine coverage by implementing additional efforts (e.g., Maine and Vermont). ^†^ Active providers included from 50 states and District of Columbia among jurisdiction partners, 21 pharmacy partners, and three federal entity partners (Health Resources & Services Administration, U.S. Department of Defense, and Veterans Health Administration) within those geographic areas. ^§^ SVI is composed of ranks from lowest (0) to highest (1) vulnerability. Rank cutoffs of 0–0.5 for low SVI and >0.5–1 for high SVI were used. In low SVI areas, 89% of children aged 5–11 years lived within 5 miles (8 km) of an active pediatric COVID-19 provider at 4 weeks after program launch. https://www.atsdr.cdc.gov/placeandhealth/svi/index.html

First dose coverage at 4 weeks after launch was 15.0% (10.5% and 17.5% in high and low SVI areas, respectively; RR = 0.68; 95% CI = 0.60–0.78) and at 11 weeks after launch was 27.7% (21.2% and 29.0% in high and low SVI areas, respectively; RR = 0.76; 95% CI = 0.68–0.84). Overall series completion at 11 weeks after launch was 19.1% (13.7% and 21.7% in high and low SVI areas, respectively; RR = 0.67; 95% CI = 0.58–0.77). Among all provider types, FRPP providers administered the highest percentage of all doses in both high SVI areas (48.7%) and low SVI areas (44.4%) (Supplementary Table 3, https://stacks.cdc.gov/view/cdc/114935).

## Discussion

To maximize pediatric vaccination opportunities, federal, state, local, and pharmacy partners developed a robust network of providers trained to serve pediatric populations and best manage the vaccine given product and supply considerations, with particular attention focused on ensuring access in the most underserved communities at risk for COVID-19–related illness and death.^¶¶¶¶^ By 4 weeks after program launch, an active COVID-19 vaccine provider was within 5 miles (8 km) of the residence of >90% of children aged 5–11 years. An estimated 27.7% of all children aged 5–11 years received a first dose of COVID-19 vaccine by 11 weeks after the program began, similar to the coverage trends reached after launch of the COVID-19 vaccination program for persons aged 12–15 years (*1*). At 11 weeks, despite 54.0% of vaccine providers being in high SVI areas, the series completion rate was approximately 33.0% lower in high than in low SVI areas, underscoring the importance of strengthening strategies (e.g., education, culturally and linguistically relevant outreach, and engagement of trusted providers) to improve vaccination coverage in these communities (*2*).

The expansion of legal authorities for the COVID-19 emergency response***** to allow pharmacists to vaccinate children and adolescents aged 3–18 years helped increase available providers and vaccine access for children aged 5–11 years. Pharmacy providers were critical in addressing high initial demand for COVID-19 vaccine among this age group, including during evenings, weekends, and over holidays, when other providers might be less available. Pharmacists also played a larger role in provision of COVID-19 vaccine to children aged 5–11 years compared with administration of routine vaccines: 46.4% of all COVID-19 pediatric vaccine doses were administered by pharmacy partners, whereas 12.3% of pediatric seasonal influenza vaccine doses were administered to children aged 5–12 years in pharmacies during 2020–21 (*3*). Pharmacies might also be important for vaccination of children aged 3–4 years if vaccine becomes available for this age group.

Likely contributors to low vaccination coverage include vaccine hesitancy among parents and caregivers and potential need for alternative convenient, trusted vaccine access points (*2*). With pediatric COVID-19 vaccine readily available in most communities, ongoing strategies to improve coverage could focus on improving vaccine confidence among caregivers through provision of information from trusted messengers, such as faith and community leaders, about the impact of COVID-19 among children (*4*–*7*) and the safety and effectiveness of COVID-19 vaccination (*8*,*9*) in culturally relevant and accessible formats to address community-level concerns (*10*).

The findings in this report are subject to at least four limitations. First, SVI metrics do not include all population characteristics that could be used to identify disparities and are measured at the county level rather than a lower administrative level such as zip code. Second, analyses of vaccine administration data were at the recipient level, with approximately 12% of data missing or suppressed because of small administration numbers, possibly having a larger effect on high SVI areas and potentially underestimating coverage in these areas. Third, spatial analysis does not consider ability to travel to the site using established transportation infrastructure, which could over- or underestimate accessibility. Finally, some private practice providers might not offer vaccine to children not already established as patients in their practice, resulting in overestimates of provider accessibility.

Initial vaccine distribution for children aged 5–11 years successfully provided vaccination opportunities within 5 miles (8 km) of most children, with 54.0% of providers located in high SVI areas. COVID-19 first-dose vaccination coverage rates were low, particularly in high SVI areas, but showed improvement over time: at 4 weeks after the program launch, first-dose vaccination coverage was 32.0% lower in children in high than in low SVI counties, and at 11 weeks after the program launch, this gap between high and low SVI area coverage was reduced to 24.0%. Ongoing efforts are critical to improving vaccination coverage among all children aged 5–11 years and reducing coverage disparities. Experiences gained through this program can be used to guide COVID-19 vaccine planning for children aged <5 years pending expansion of COVID-19 vaccine recommendations for this age group. Specifically, planning could consider vaccine supply, vaccine formulation (i.e., shelf life or doses per vial), fewer vaccinations provided in pharmacies, preferred vaccination locations in communities, community risk, vulnerability, and geography.

**Summary**

**What is already known about this topic?**

Successful vaccination coverage requires access to vaccine and uptake. COVID-19 vaccination coverage in children is low.

**What is added by this report?**

At 11 weeks after launch of the pediatric COVID-19 vaccination program, 92% of children aged 5–11 years lived within 5 miles (8 km) of a pediatric vaccine provider; 44% of providers were pharmacies. COVID-19 first-dose vaccination coverage rates were low, particularly in high social vulnerability index (SVI) areas, but improved over time.

**What are the implications for public health practice?**

Broad vaccine access should be maintained while critical outreach efforts continue to improve vaccine coverage among children aged 5–11 years, especially in high SVI areas. If COVID-19 vaccine is recommended for children aged <5 years, similar efforts to strategically maximize access and coverage might be considered.

## Footnotes

* These authors contributed equally to this report.

†
https://www.fda.gov/news-events/press-announcements/fda-authorizes-pfizer-biontech-covid-19-vaccine-emergency-use-children-5-through-11-years-age

§ Fifteen SVI indicators: 1) percentage of persons with incomes below poverty threshold, 2) percentage of civilian population (aged ≥16 years) who is unemployed, 3) per capita income, 4) percentage of persons aged ≥25 years with no high school diploma, 5) percentage of persons aged ≥65 years, 6) percentage of persons aged ≤17 years, 7) percentage of civilian noninstitutionalized population with a disability, 8) percentage of single-parent households with children aged <18 years, 9) percentage of persons who are racial/ethnic minorities (i.e., all persons except those who are non-Hispanic White), 10) percentage of persons aged ≥5 years who speak English “less than well,” 11) percentage of housing in structures with ≥10 units (multiunit housing), 12) percentage of housing structures that are mobile homes, 13) percentage of households with more persons than rooms (crowding), 14) percentage of households with no vehicle available, and 15) percentage of persons in group quarters. The 15 indicators are categorized into four themes: 1) socioeconomic status (indicators 1–4), 2) household composition and disability (indicators 5–8), 3) racial/ethnic minority status and language (indicators 9 and 10), and 4) housing type and transportation (indicators 11–15). Overall SVI includes all 15 indicators as a composite measure and a final score is ranked from lowest (0) to highest (1) vulnerability. https://www.atsdr.cdc.gov/placeandhealth/svi/index.html

¶ VFC is a federally funded program that provides routine childhood vaccines through VFC participating providers (i.e., private physicians’ offices and public health clinics) at no cost to children who might not otherwise be vaccinated because of inability to pay. Federally Qualified Health Centers, the Rural Health Clinics program, and state and local health departments are also VFC/primary care providers. https://www.cdc.gov/vaccines/programs/vfc/index.html

** Jurisdiction partners consisted of 62 states, territories, and cities, including the 50 states, District of Columbia, three cities (Chicago, Illinois; New York, New York; Philadelphia, Pennsylvania), five U.S. territories (American Samoa, Guam, Northern Mariana Islands, Puerto Rico, and U.S. Virgin Islands), and three freely associated states (Federated States of Micronesia, Marshall Islands, and Palau).

†† Pharmacy partners include 21 retail and independent pharmacy networks across the United States. https://www.cdc.gov/vaccines/covid-19/retail-pharmacy-program/index.html

§§ Federal entity partners that received direct allocations of COVID-19 pediatric vaccine include U.S. Department of Defense, U.S. Department of State, Indian Health Service, Veterans Health Administration, and the Health Resources & Services Administration. A complete list of federal entity partners receiving direct allocation of COVID-19 vaccine can be found at https://www.cdc.gov/vaccines/imz-managers/downloads/Covid-19-Vaccination-Program-Interim_Playbook.pdf.

¶¶ The new pediatric formulation was packaged in 10-dose vials with a minimum order requirement of 100 doses (300 doses during the first week) and has a shorter shelf life (6 months) than the adult formulation (9 months), risking higher wastage. Partners considered the following in their site selection: provider type, site training and ability to vaccinate younger age groups, geographic access, provider ability to store and administer vaccine given limited shelf life, provider throughput, and community level demand to minimize wastage of initial limited supply. Initially 15 million doses were made available to partners to order and by 11 weeks 39 million doses were made available. https://www.cdc.gov/vaccines/covid-19/info-by-product/pfizer/pfizer-bioNTech-children-adolescents.html

***
https://www.cdc.gov/coronavirus/2019-ncov/vaccines/distributing/about-vaccine-data.html

††† https://www.census.gov/programs-surveys/popest.html (Accessed January 28, 2022).

§§§ https://www.worldpop.org/project/categories?id=3 (Accessed January 28, 2022).

¶¶¶ Geospatial analyses to produce maps were done only on the geographic boundaries of the 50 states and District of Columbia using Census tract–level SVI with populations aggregated at the state level. WorldPop age-specific raster files from 2020 using 5-mile [8-km] buffer zones around active provider coordinates were used to estimate pediatric proximity and coverage, overall and limited to children residing in high SVI areas.

**** https://covid.cdc.gov/covid-data-tracker/#vaccination-equity (Accessed January 28, 2022).

†††† State fixed effects and robust variance were also used.

§§§§ 45 C.F.R. part 46.102(l)(2), 21 C.F.R. part 56; 42 U.S.C. Sect. 241(d); 5 U.S.C. Sect. 552a; 44 U.S.C. Sect. 3501 et seq.

¶¶¶¶ A successful vaccination program requires substantial planning, including vaccine development and evaluation, adequate vaccine production and supply to meet anticipated demand, a predictable and stable network of administration sites, public demand and trust, and strategic vaccine delivery to best reach the eligible population. Like other COVID-19 vaccine program launches (i.e., initiation of adolescent vaccination and booster vaccination), sites expected a higher demand during the initial weeks of the program. Site selection balanced vaccine access with expected demand to avoid distributing supply across too many providers, potentially decreasing vaccination opportunities at high demand sites, and increasing vaccine wastage at low demand sites. COVID-19 vaccine provider sites were expected to have trained staff members specialized in vaccinating children. Providers were asked to consider vial size (10 doses) and 6-hour time frame when scheduling children for vaccination, especially early in the program to minimize waste and optimize use of supply. https://www.cdc.gov/vaccines/covid-19/downloads/Pediatric-Planning-Guide.pdf

***** On August 24, 2020, the Public Readiness and Emergency Preparedness Act amendment resulted in the HHS Secretary amending the Declaration to identify state-licensed pharmacists (and pharmacy interns acting under their supervision if the pharmacy intern is licensed or registered by their state board of pharmacy) as qualified persons under section 247d–6d(i)(8)(B) to administer vaccine to persons aged 3–18 years. This act covers all Advisory Committee on Immunization Practices recommended vaccines. These requirements are consistent with those in many states that permit licensed pharmacists to administer vaccines to children and adolescents. Expansion was in response to an identified decline in routine pediatric vaccine coverage indicating that U.S. children and adolescents and their communities face increased risks for outbreaks of vaccine-preventable diseases. CDC reports suggested that decreases in rates of routine childhood vaccinations were because of changes in health care access, decrease in well-child visits, increased physical distancing, and other COVID–19 prevention strategies. https://www.govinfo.gov/content/pkg/FR-2020-08-24/pdf/2020-18542.pdf
